# S121. JUMPING TO CONCLUSIONS, SOCIAL COGNITION AND METACOGNITION IN PEOPLE WITH A RECENT-ONSET OF PSYCHOSIS

**DOI:** 10.1093/schbul/sby018.908

**Published:** 2018-04-01

**Authors:** Raquel López-Carrilero, Luciana Díaz, Esther Pousa, Eva Grasa, Ana Barajas, Maria Luisa Barrigon, Fermin Gonzalez Higueras, Esther Lorente, Jordi Cid, Isabel Ruiz, Irene Birules, Susana Ochoa

**Affiliations:** 1 Parc Sanitari Sant Joan de Deu; 2 Hospital Moyano; 3 Parc Salut Mar; 4 IIB-Hospital Santa Creu I Sant Pau; 5 Centro de Higiene Mental Les Corts, Research Unit; 6 Fundación Jiménez Díaz Hospital; 7 Servicio Andaluz de Salud Jaen; 8 Hospital Clínico Universitario Valencia; 9 Salut Mental i Addiccions IAS Girona; 10 Servicio Andaluz Salud Málaga; 11 Parc Sanitari Sant Joan de Déu

## Abstract

**Background:**

The reasoning bias of jumping to conclusions (JTC) consists of a tendency to have an impaired decision process in which assumptions are made having little information. JTC is one of the most widely studied cognitive biases in psychosis (Freeman, 2007; Garety and Freeman, 1999; Moritz and Woodward, 2005) due to its higher prevalence in people with psychosis in comparison to healthy participants as found by So et al. (2016) in her meta-analyses and by Dudley et al. (2016) in his systematic review and meta-analysis. Social cognition(sc) is the best defined as a set of neurocognitive processes related to understanding, recognizing, processing, and appropriately using social stimuli in one′s environment (Adolphs, 1999). The domains are: emotion processing, theory of mind, attributional style and social perception.

**Methods:**

One hundred and twenty patients with a recent onset of a psychotic disorder were assessed. Jumping to conclusions (JTC) was assessed with the beads task in which the subject must take a decision regarding the probability of the extracted bead belonging to one of two jars. In task 1 a jar is presented with a ratio of 85% black beads and 15% orange beats and another jar with inverse proportion. Task 2 is the same but the probability is 60%-40%. Task 3 has the same probability 60%-40& but instead of beads, the jars contain negative and positive adjectives. JTC was considered as taking a decision after extracting one or two beads (Brett-Jones et al. 1987). A battery of questionnaires regarding social cognition was included: The Hinting Task, was used to assess ToM. (Corcoran et al. 1995; Gil et al. 2012); Emotional perception was assessed with the Emotional Recognition Test Faces (Baron-Cohen et al. 1997), and the attributional style was assessed with the Internal, Personal and Situational Attributions Questionnaire (IPSAQ; Kinderman & Bentall, 1996). Irrational beliefs were assessed with the irrational Belief Test (TCI; Calvete & Cardeñoso, 2001). The scale is composed of ten subscales: needing acceptance from others, high expectations, guilt, intolerance to frustration, worry and anxiety, emotional irresponsibility, avoidance of problems, dependence, helplessness, and perfectionism.

**Results:**

Patients who performed JTC in Task 1 scored higher levels of worry and anxiety (p=0.026), perfectionism (p=0.01) and internal attribution for negative events (p=0.034) and lower in externalizing bias (p=0.042) and emotional recognition (p=0.042). Patients who performed JTC in Task 2 scored higher levels of worry and anxiety (p=0.016) and lower in emotional recognition (p=0.031). And finally, patients who performed JTC in Task 3 scored higher in internal attribution for negative events (p=0.029) and worry and anxiety (p=0.002) whereas that lower in situational attribution for negative events (p=0.004) and emotional recognition (p=0.017). ToM is not related with any of the JTC tasks.

**Discussion:**

JTC is related to some aspects of social cognition, specifically with attributional style and emotional recognition. Moreover, JTC is related with perfectionism and worry and anxiety irrational beliefs.

